# Discrepancy between Subjective and Objective Measurements for the Evaluation of Medication Adherence—A Cross-Sectional Study in Patients with Cardiovascular Diseases

**DOI:** 10.3390/pharmacy12050153

**Published:** 2024-10-06

**Authors:** Motoyasu Miyazaki, Hitomi Hirata, Satoko Takaki, Momoko Misaki, Yukako Mori, Kaoko Tokura, Natsuki Sato, Akio Nakashima, Atsuko Yanagida, Isa Okajima, Hidenori Urata, Osamu Imakyure

**Affiliations:** 1 Department of Pharmacy, Fukuoka University Chikushi Hospital, Fukuoka 818-8502, Japan; h.hirata.cd@adm.fukuoka-u.ac.jp (H.H.); takaki102@fukuoka-u.ac.jp (S.T.); mmisaki@fukuoka-u.ac.jp (M.M.); yamaguchi320@fukuoka-u.ac.jp (Y.M.); ktokura@fukuoka-u.ac.jp (K.T.); fupp160157@gmail.com (N.S.); anakashima@fukuoka-u.ac.jp (A.N.); imakyure@fukuoka-u.ac.jp (O.I.); 2 Pharmaceutical and Health Care Management, Faculty of Pharmaceutical Sciences, Fukuoka University, Fukuoka 814-0180, Japan; 3 Graduate School of Humanities and Life Sciences, Tokyo Kasei University, Tokyo 173-8602, Japan; atk.yngd@gmail.com; 4 Department of Psychological Counseling, Faculty of Humanities, Tokyo Kasei University, Tokyo 173-8602, Japan; okajima-i@tokyo-kasei.ac.jp; 5 Department of Cardiovascular Diseases, Fukuoka University Chikushi Hospital, Fukuoka 818-8502, Japan; jyunurata@gmail.com

**Keywords:** medication adherence, medication compliance, cardiovascular diseases, questionnaire survey

## Abstract

Medication adherence is important for the appropriate drug-based treatment in patients with chronic diseases, especially those with cardiovascular diseases (CVDs). The purpose of the present study was to evaluate medication adherence among patients with CVDs using subjective and objective measurements. We enrolled outpatients who visited Fukuoka University Chikushi Hospital from June to December 2022. As a subjective measurement, we used a self-reported questionnaire developed by Ueno et al., which consists of 12 questionnaire items grouped into the following four domains: medication compliance (subjective compliance), collaboration with health care providers (collaboration), willingness to access and use information about medication (willingness), and acceptance to take medication and how taking medication fits a patient’s lifestyle (acceptance). The pill counting method was used as an objective measurement to calculate the medication adherence rate; Poor Adherence was defined as a medication adherence rate of <100%. Ninety-four patients were analyzed. No statistically significant differences were observed between the patients in the Good and Poor Adherence groups classified by pill counting, an objective indicator; in the subjective evaluation index Ueno scale scores of subjective compliance, collaboration, willingness, and acceptance domains; and in the total score. A multivariate analysis revealed that obesity (odds ratio, 3.527; 95% confidence interval, 1.387–9.423; *p* = 0.008) was an independent factor associated with Poor Adherence. In conclusion, we found a discrepancy between subjective and objective measurements for the evaluation of medication adherence. Furthermore, obesity was an independent factor associated with poor medication adherence assessed by the pill counting method; thus, patients with CVD and obesity require a careful follow-up.

## 1. Introduction

According to the Annual Report on the Ageing Society FY2022, the population aged 65 and older comprises 36.21 million individuals, and the percentage of the elderly in Japan is 28.9% [1]. Moreover, the percentage of elderly people in Japan is expected to reach 38.4% by 2065, and it is estimated that approximately 1 in 2.6 people will be over 65 years of age at that time. As the population ages, the number of patients with chronic diseases is increasing, and drug-based treatment is expected to play an increasingly important role in the future. According to the latest data from the Ministry of Health, Labour, and Welfare of Japan (2023), cardiovascular diseases account for 14.7% of all deaths and rank as the second leading cause of death, following cancer, which accounts for 24.3% [2]. Medication adherence is important for the appropriate drug-based treatment of patients with chronic diseases, especially those with cardiovascular diseases (CVDs) [3,4]; however, medication nonadherence is a common phenomenon worldwide, leading to poor patient outcomes and having a substantial impact on health care costs [5,6].

Generally, the measurements of medication adherence are classified by the WHO as subjective and objective measurements [7]. Subjective measurements include self-reported questionnaires, such as the eight-item Morisky Medication Adherence Scale [8], the Medication Adherence Rating Scale [9], and other questionnaires [10]. Objective measurements are classified into two types: indirect and direct methods. Indirect methods include pill counting, electronic monitoring, and formulations such as the proportion of days covered or the medication possession ratio [11,12], whereas direct methods include the measurement of drug concentrations in body fluids, such as the blood or urine [13]. However, there is no gold standard method to measure medication adherence.

To appropriate the self-administration of medicines in daily life, not only the patients’ understanding of the drug-dosing regimen, including time, dosage, and interval of medication intake, but also the selection of medicines that suits their lifestyle, an understanding of the necessity of taking the medication, a relationship of trust between the patients and medical professionals, and the patient’s motivation for taking the drug are important [7]. Ueno et al. created a self-reported 12-item medication adherence measuring scale that considers these concepts; those authors reported its reliability and validity for Japanese patients with chronic disease, including heart disease and coronary risk factors [14]. In addition, using this scale, Ueno et al. performed a survey of home-dwelling elderly people who required drug treatment, and found that functional health literacy, communicative health literacy, and good communication with doctors were significantly associated with a high level of medication adherence [15]. Although the Ueno scale is a patient-based subjective evaluation measurement of medication adherence [14,15], it has not exhibited a relationship with the medication adherence rate calculated from the actual number of medications taken (i.e., pill count), which is an objective measurement.

The main purpose of this study was to evaluate medication adherence in patients with CVDs using the Ueno scale and to assess the medication adherence rate via pill counting; moreover, the relationship between these parameters was also examined. The secondary purpose of the study was to explore the demographic and clinical factors associated with the medication adherence rate.

## 2. Materials and Methods

### 2.1. Study Participants

In this study, we enrolled 202 outpatients who visited the Department of Cardiovascular Diseases at Fukuoka University Chikushi Hospital, Fukuoka Japan, from June to December 2022. To conduct a pill counting survey, patients who had visited the clinic at least twice and whose visit intervals were between 30 and 180 days were included. Patients who did not provide consent, those unable to manage their medication independently, those without medications, and those who had an interval of more than 180 days between two visits were excluded from the study. To minimize the selection bias (i.e., to remove the effect of physician interactions with patients), we only included patients who were followed by a single physician (H.U.). The study was conducted in accordance with the Declaration of Helsinki and was approved by the Fukuoka University Medical Ethics Review Board (C22-06-001). Written informed consent was obtained from all subjects enrolled in the study.

### 2.2. Patient Characteristics

We investigated the clinical characteristics of the patients, including age, gender, history of side effects and allergies, medical history, hospitalization history, concomitant medications, and the presence of one-dose packaging (ODP), using their electronic medical records. ODP is a medication packaging system used in Japanese pharmacies, where prescribed medications are divided into individual doses and packaged together. This is especially helpful when patients need to take multiple medications, as it simplifies their medication schedule by combining all the medications they need to take at a given time into one packet. Polypharmacy was defined as the use of six or more medications [16]. In addition, we assessed height, weight, body mass index (BMI), smoking history, alcohol consumption habits, sleep quality, exercise habits, activities of daily living (ADLs), prescriptions from another hospital, living status (alone or not), place of residence, and employment status using a self-administered questionnaire. The questions for patients and the definitions of each item are shown in Table S1. Obesity was defined as a BMI > 25 kg/m^2^.

### 2.3. Evaluation of Medication Adherence Using a Subjective Measurement

We used the 12-item Medication Adherence Scale developed by Ueno et al. (referred to as the “Ueno scale” in this manuscript), the reliability and validity of which have been validated (Cronbach’s coefficient alpha was 0.78) [14,15]. This study utilized a self-administered paper-based questionnaire written in Japanese. The Ueno scale is a subjective tool for the assessment of patients’ medication adherence that consists of 12 questionnaire items grouped into the following four domains: medication compliance (referred to as “subjective compliance”), collaboration with health care providers (referred to as “collaboration”), willingness to access and use information about medication (referred to as “willingness”), and acceptance to take medication and how taking medication fits the patient’s lifestyle (referred to as “acceptance”). Each item is rated on a 1–5-point Likert scale, and each domain is scored on a total of 3–15-point scale. The overall medication adherence score, which ranged from 12 to 60, was also calculated by adding the scores on all 12 items of the Ueno scale, with higher scores indicating a better medication adherence. The domain of medication compliance was scored based on the pre-hospitalization experience, whereas the remaining domains were scored based on the perspective and attitude toward the treatment and medications being administered at the time of the questionnaire response. Permission to use this scale was obtained from the developers of the original scale.

### 2.4. Assessment of the Medication Adherence Rate Using Pill Counting

We used the pill counting method as an objective measurement, which verified the remaining medication. The period between the first and second visits was set between 30 and 180 days. The medication adherence rate was calculated using the following formula: Medication adherence rate (%) = (number of prescribed medications−number of remaining medications)/number of prescribed medications × 100. Number of prescribed medications was defined as the quantity of medications that should have been taken from the first visit to the second visit. Number of remaining medications was defined as the medications remaining on the second visit.

### 2.5. Statistical Analysis

For the analysis of categorical data, we employed either the chi-squared test or Fisher’s exact test, whereas the Mann–Whitney *U* test was used to analyze the numerical data. In all cases, *p* < 0.05 was considered statistically significant.

“Good Adherence” was defined as a medication adherence rate of 100%, whereas “Poor Adherence” was defined as a medication adherence rate <100%. We performed a comparison between the two groups regarding the patient characteristics and the score on the Ueno scale (univariate analysis). The factors that were likely to be associated with Poor Adherence in the univariate analysis (*p* < 0.1) were applied as independent variables in the multivariate logistic regression analysis, using “Good Adherence” and “Poor Adherence” as the dependent variables. Statistical analyses were performed using the JMP 10.0.2 software.

## 3. Results

### 3.1. Demographic and Clinical Characteristics

Of the 202 outpatients who visited our hospital during the study period, 108 patients who met the exclusion criteria were excluded from the study, leaving 94 patients for analysis. The reasons for exclusions were as follows: 6 patients did not provide consent, 4 patients were unable to manage their medication on their own, 88 patients were not taking their medication, and 10 patients had an interval of more than 180 days between their two visits.

The demographic and clinical characteristics of the 94 patients are listed in Table 1. Among these 94 patients, 52 individuals were men, and the median age was 74 years. The median number of medications per patient was 4, and 16 patients (17.0%) used ODP. Moreover, approximately half of the patients (*n* = 49 individuals (52.1%)) had a medication adherence rate of 100%. A medication adherence rate of 90–99% was observed in 38 patients (40.4%), whereas five patients (5.3%) had medication adherence rates of 80–89% and two patients (2.1%) had a medication adherence rate of <80%.

**Figure 1:**
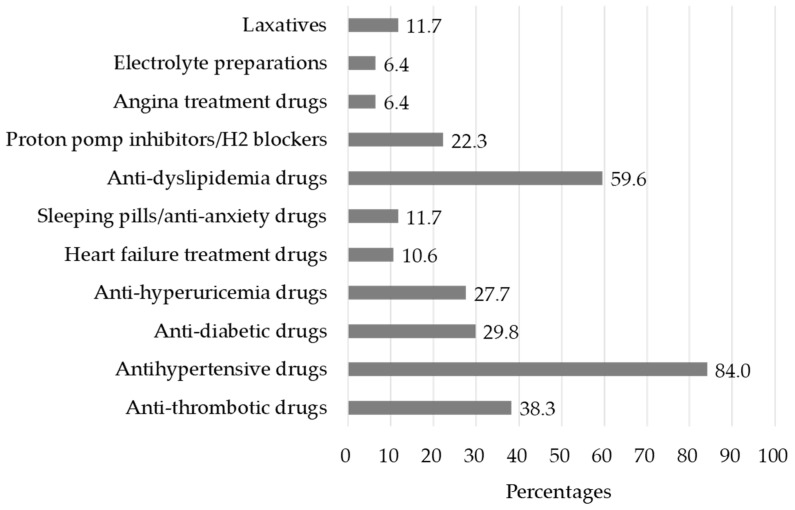
Types of medications used by the patients.

The types of medications used by the patients are reported in Figure 1. Antihypertensive drugs were the most prevalent drugs, as they were taken by 79 patients (84.0%), followed by anti-dyslipidemia drugs in 56 patients (59.6%), anti-thrombotic drugs in 36 patients (38.3%), anti-diabetic drugs in 28 patients (29.8%), anti-hyperuricemia drugs in 26 patients (27.7%), and PPI or H2 blockers in 21 patients (22.3%).

### 3.2. Evaluation of Medication Adherence Using the Ueno Scale

The median score values (interquartile ranges) obtained for the subjective compliance, collaboration, willingness, and acceptance domains of the Ueno scale were 15 (15–15), 12 (10–14), 10 (7–12), and 13 (12–15), respectively. No statistically significant differences were observed between the patients in the Good and Poor Adherence groups in the subjective compliance, collaboration, willingness, and acceptance domains, and in the total score on the Ueno scale (Table 2).

### 3.3. Factors Associated with Poor Adherence

Table 3 provides a comparison of the demographic and clinical characteristics between the Good and Poor Adherence groups. The proportion of patients with obesity and smoking history was significantly higher in the Poor Adherence group (48.9% and 53.3%, respectively) than it was in the Good Adherence group (24.5% and 32.7%, respectively), with *p* values of 0.014 and 0.043, respectively. The rate of the patients living alone was higher in the Good Adherence group compared to the Poor Adherence group (20.4% vs. 6.7%), while those with good ADLs and those with angina treatment drug usage were higher in the Poor Adherence group (62.2% and 11.1%, respectively) compared to the Good Adherence group (44.9% and 2.0%, respectively). However, these differences did not reach statistical significance.

A multivariate logistic regression analysis revealed that obesity [odds ratio (OR), 3.527; 95% confidence interval (CI), 1.387–9.423; *p* = 0.008] was an independent factor associated with Poor Adherence (Table 4). Smoking history (OR, 2.442; 95% CI, 0.985–6.277; *p* = 0.054) and angina treatment drug usage (OR, 7.393; 95% CI, 0.982–154.3; *p* = 0.052) were associated with Poor Adherence, though the *p* values did not reach statistical significance.

## 4. Discussion

In this study, we examined the relationship between the scores on the Ueno Scale and medication adherence rate by pill counting, and found that no relationship was detected between medication adherence rate by pill counting and the scores on the subjective compliance, collaboration, willingness, and acceptance domains, as well as the total score on the Ueno scale. Furthermore, because medication adherence in patients with CVDs is an important factor related to clinical outcomes [17], we defined Poor Adherence as an objective medication adherence rate of <100%, and analyzed factors related to it in this study. Obesity was an independent factor associated with poor objective medication adherence in the multivariate logistic regression analysis.

Medication adherence is defined as “the process by which patients take their medications as prescribed” and is divided into three inter-related yet distinct stages of adherence: initiation, implementation, and persistence [18]. In this study, we focused on patients in the “implementation” stage. In general, adherence levels vary according to the measurement methods, with subjective methods being more prone to provide underestimations of the prevalence of Poor Adherence [19]. Here, subjective evaluation using the Ueno scale tended to provide higher scores compared with objective evaluation via pill counting regarding “medication compliance,” which is consistent with previous reports.

Furthermore, there is no consensus regarding the cutoff value that defines optimal adherence to medications for CVDs, although the 80% threshold appears to be the most widely used criterion in clinical research [20,21]. Here, only 2.1% of the patients had a medication adherence rate of <80%, as an objective indicator. Mathews et al. evaluated inter-hospital variation in the adherence rates to secondary prevention medications and demonstrated that hospitals with a high adherence rate were typically larger and academic centers, which may provide systematic and comprehensive support for improving adherence after discharge [22]. This study also targeted patients who were followed at a university hospital; thus, it is possible that patients who had undergone interventions and received education that would increase adherence were selected.

Medication nonadherence leads to poor outcomes, which substantially increases health care service usage and health care costs; therefore, interventions that improve medication adherence may not only improve the quality of life of patients, but also reduce the clinical and economic burden on the health care system [5,6,22]. Bahit et al. suggested that adherence to medications in secondary prevention after acute coronary syndromes is affected by various factors, including psychological-, provider-, social-, clinical-, and system-based factors [17]. The social factors associated with medication adherence include the level of education and overall health literacy [17]. Here, obesity was associated with poor objective medication adherence, and smoking history exhibited a similar tendency. Michou et al. reported that excess body weight was associated with a low health literacy [23]. Stewart et al. also reported that, among racially/ethnically diverse smokers with a low socioeconomic status, a lower health literacy was associated with higher nicotine dependence, more positive and less negative smoking outcome expectations, less knowledge about the health risks of smoking, and lower risk perceptions [24]. Therefore, as described in the Results section of the present study, in patients with obesity or a smoking history, it is possible that a decrease in health literacy causes a decrease in medication adherence.

The present study had several limitations. First, this was a single-center study with a relatively small cohort, which may have led to a selection bias. The participants may have been highly motivated regarding medications, as reflected by the high medication adherence rate. Second, although patient-related factors and disease- or clinical-related factors were investigated as being related to medication adherence, factors related to the health care system and social factors were been investigated in this context. Third, the medication or medical history of the targeted patients could not be reviewed. We have previously shown that, in patients with atrial fibrillation, a longer history of taking direct oral anticoagulants implies a lower medication adherence [25]. Fourth, although we used a pill counting method to assess objective medication adherence, there are concerns that medication adherence may be overestimated due to factors such as patients discarding medication at home. The usefulness of the Medication Event Monitoring System and electronic monitoring devices have also been reported as an objective indicator [26], and further research using a model that overcomes these limitations is required to elucidate this issue fully. Finally, we used the Ueno scale as a method to evaluate subjective medication adherence. The total score range of the Ueno scale is 12 to 60 points, but it is unclear what score indicates Good medication adherence. Future prospective studies are needed to examine how this may impact clinical outcomes.

## 5. Conclusions

In conclusion, we observed a discrepancy between medication adherence evaluations using subjective and objective measurements. In clinical practice, it is important to establish a trusting relationship with patients and to use both subjective and objective indicators to monitor patients’ adherence. In addition, obesity was an independent factor associated with poor medication adherence assessed by pill counting. While careful follow-up is necessary for all patients, those with chronic conditions, particularly CVDs associated with obesity, require especially diligent monitoring.

## Figures and Tables

**Table 1:** Demographic and clinical characteristics of the 94 patients included in the analysis.

Characteristics	*n* (%)
Age (years) ^a^	74 (67–81)
Male/Female gender	52 (55.3)/42 (44.7)
Obesity	34 (36.2)
Smoking history	40 (42.6)
Alcohol consumption habits	43 (45.7)
Good sleep	64 (68.1)
Exercise habits	64 (68.1)
Good ADLs	50 (53.2)
History of side effects or allergies	17 (18.1)
Hospitalization history	60 (63.8)
Prescription from another hospital	54 (57.4)
Living alone	13 (13.8)
Employment	35 (37.2)
Number of medications ^a^	4 (2–7)
ODP	16 (17.0)
Medication adherence rate = 100% (Good Adherence)	49 (52.1)

ADL, activities of daily living; ODP, one-dose package. ^a^: median (interquartile range).

**Table 2:** Comparison of the scores on the Ueno scale between the Good and Poor Adherence groups.

Ueno Scale	Good Adherence (*n* = 49)	Poor Adherence (*n* = 45)	*p* Value
Subjective compliance	15 (15–15)	15 (14.5–15)	0.083
Collaboration	12 (10–15)	12 (9.5–14)	0.250
Willingness	10 (7–12.5)	10 (7–12)	0.918
Acceptance	13 (12–15)	13 (12–14)	0.459
Total	51 (44–56)	50 (45–54)	0.426

The scores are reported as the median (interquartile range).

**Table 3:** Comparisons of demographic and clinical characteristics between the Good and Poor Adherence groups.

Characteristics	Good Adherence (*n* = 49)	Poor Adherence (*n* = 45)	*p* Value
Age (years) ^a^	75 (64–81)	74 (67–78)	0.8143
Gender			0.3812
Male	25 (51.0)	27 (60.0)	
Female	24 (49.0)	18 (40.0)	
Obesity	12 (24.5)	22 (48.9)	**0.014**
Smoking history	16 (32.7)	24 (53.3)	**0.043**
Alcohol consumption habits	22 (44.9)	21 (46.7)	0.864
Good sleep	34 (69.4)	30 (66.7)	0.777
Exercise habits	32 (65.3)	32 (71.1)	0.546
Good ADLs	22 (44.9)	28 (62.2)	0.093
History of side effects or allergies	9 (18.4)	8 (17.8)	0.941
Hospitalization history	31 (63.3)	29 (64.4)	0.905
Prescription from another hospital	28 (57.1)	26 (57.8)	0.950
Living alone	10 (20.4)	3 (6.7)	0.074
Employment	18 (36.7)	17 (37.8)	0.917
Number of medications ^a^	4 (2–6)	4 (3–7)	0.506
Polypharmacy	16 (32.7)	15 (33.3)	0.944
ODP	9 (18.4)	7 (15.6)	0.717
Types of medications			
Anti-thrombotic drugs	17 (34.7)	19 (42.2)	0.453
Antihypertensive drugs	44 (89.8)	35 (77.8)	0.112
Anti-diabetic drugs	11 (22.4)	17 (37.8)	0.105
Diuretic drugs	4 (8.2)	2 (4.4)	0.461
Anti-hyperuricemia drugs	15 (30.6)	11 (24.4)	0.504
Heart failure treatment drugs	6 (12.2)	4 (8.9)	0.598
Sleeping pills/anti-anxiety drugs	7 (14.2)	4 (8.9)	0.416
Anti-dyslipidemia drugs	28 (57.1)	28 (62.2)	0.616
Proton pomp inhibitors/H2 blockers	10 (20.4)	11 (24.4)	0.639
Angina treatment drugs	1 (2.0)	5 (11.1)	0.072
Electrolyte preparations	2 (4.1)	4 (8.9)	0.341
Laxatives	5 (10.2)	6 (13.3)	0.637

ADLs, activities of daily living; ODP, one-dose package. ^a^: median (interquartile range). **Bold** indicates statistical significance (*p* < 0.05).

**Table 4:** Factors associated with Poor Adherence in the multivariate logistic regression analysis.

Characteristics	Odds Ratio	95% Confidence Interval	*p* Value
Obesity	3.527	1.387–9.423	**0.008**
Smoking history	2.442	0.985–6.277	0.054
Good ADLs	1.341	0.501–3.625	0.559
Living alone	0.336	0.062–1.430	0.144
Angina treatment drug usage	7.393	0.982–154.3	0.052
Subjective compliance (Ueno scale) ^a^	0.867	0.476–1.468	0.597

ADLs, activities of daily living. **Bold** indicates statistical significance (*p* < 0.05). ^a^: OR per 1 point increase.

## Data Availability

Data are contained within this article.

## Supplementary Materials

The following supporting information can be downloaded at: https://www.mdpi.com/article/10.3390/pharmacy12050153/s1, Table S1: The questions for patients and the definitions of each item.
